# Gestational age-based outcomes of neonates with Down syndrome in the neonatal intensive care unit (NICU): review of pediatric health information system (PHIS) database

**DOI:** 10.1038/s41372-025-02384-0

**Published:** 2025-08-12

**Authors:** Emily A. Messick, Stephen A. Hart, Julie Strominger, Sara Conroy, Carl H. Backes, Clifford L. Cua

**Affiliations:** 1 Department of Pediatrics, Nationwide Children’s Hospital, Columbus, OH USA; 2 Division of Neonatology, Nationwide Children’s Hospital, Columbus, OH USA; 3 Division of Cardiology, Nationwide Children’s Hospital, Columbus, OH USA; 4 Ohio Perinatal Research Network, Nationwide Children’s Hospital, Columbus, OH USA; 5 Department of Biostatistics, The Ohio State University, Columbus, OH USA

**Keywords:** Risk factors, Aneuploidy

## Abstract

**Objective:**

To examine differences in neonatal intensive care unit (NICU) outcomes in neonates with Down syndrome (DS) by gestational age (GA) using a large national database

**Study design:**

Retrospective analysis of Pediatric Health Information System database, including neonates with DS admitted to the NICU <30 days old from 1/1/2008-12/31/2022. Neonates were stratified by GA (extremely preterm, very preterm, moderate/late preterm, term). GA-based risk differences were examined for NICU outcomes using term neonates as reference.

**Results:**

Overall mortality rate was 7% with increasing mortality as GA decreased (RD 6.1 [95% CI 4.8, 7.5], RD 25.4 [95% CI 20.5, 30.6], RD 36.8 [95% CI 27.3, 46.8] for moderate/late preterm, very preterm, extremely preterm). The overall rate of pulmonary hypertension was 23% and 14% of the cohort required a gastrostomy tube.

**Conclusion:**

Prematurity significantly increases risk of NICU mortality for neonates with DS. Rates of pulmonary hypertension and gastrostomy were high throughout all groups.

## Background

Down syndrome (DS) is the most viable and common of all trisomies, occurring in approximately 1 in 700 live births in the US [1]. Patients with DS have an increased risk for multiple congenital as well as physiologic abnormalities that may increase their overall morbidity and mortality compared to those without DS throughout their lifetime [2–13]. Risk factors for increased mortality in this population include, but are not limited to: low birth weight, presence of cardiac conditions such as atrioventricular septal defects, development of medical complications like pneumonia, and procedural variables that occurred during their hospitalization, including catheter placements [5–8, 10, 14–17].

Neonates with DS present a unique population with approximately 20% being born prematurely and 65–85% requiring neonatal intensive care unit (NICU) hospitalization [18–21]. Prematurity and DS likely increase the overall jeopardy of adverse outcomes due to their attendant risks. Earlier gestational age (GA) has been noted to be a risk factor for increased mortality in neonates with DS in previous publications [8, 18, 21, 22]. These previous publications may have been limited due to being single-center or regional evaluations or dichotomizing prematurity as yes or no. There is limited to no national data evaluating the effect of prematurity in this at-risk population.

The goal of this study was to examine GA-based differences for in-hospital morbidities and mortality in neonates with DS admitted to the NICU using a large national database.

## Methods

### Data source

Data were obtained from the Pediatric Health Information System (PHIS) of the Child Health Corporation of America (Shawnee Mission, KS). The PHIS database contains administrative, billing, and record-review data, including patient demographics (ex. sex, birth weight, GA) as recorded in the patient chart, diagnoses, medications, and procedures, from more than 45 freestanding US children’s hospitals, which account for 85% of all national freestanding children’s hospitals. To certify the comparability of charge-level data among institutions, including medications and procedures, Thompson-Reuters Healthcare (Ann Arbor, MI), the PHIS data processing partner, mapped each hospital’s daily charge codes to a common classification system, the Clinical Transaction Classification (CTC) codes. Previous studies have utilized the PHIS database for descriptive analyses of those with DS, but these publications either used NICU data not from the current era or did not specifically evaluate NICU admissions [2, 14, 23–28].

### Ethics approval and consent to participate

All methods were performed in accordance with the relevant guidelines and regulations. Because PHIS contains de-identified information, Investigational Review Board (IRB) determined that this study did not fit the definition of human subjects’ research under 45 CFR part 46.102(f); therefore, IRB evaluation was waived.

### Study population

Neonates admitted to a participating PHIS site from 1/1/2008–12/31/2022 were identified. Analysis was subset to neonates with DS admitted to the hospital at <30 days of age and with an overlapping NICU stay. Those with DS were identified using *International Classification of Diseases, Ninth Revision, Clinical Modification or International Statistical Classification of Diseases and Related Health Problems, Tenth Revision* diagnosis codes 758.0 and Q90.9, respectively. Neonates were excluded if GA was missing, less than 22 weeks, or greater than 42 weeks. If a given neonate had multiple NICU hospitalizations, data were collapsed.

### Characteristic and outcome definitions

Neonates were grouped based on completed weeks of gestation at birth according to the World Health Organization [29]: <28 weeks (extremely preterm), 28–31 (very preterm), 32–36 (moderate/late preterm), and ≥37 weeks (term). A variety of characteristics were derived, including infant sex, race, ethnicity, urbanicity, 2010 zip code-level median household income, and primary payor. Race and ethnicity were collected according to hospital-specific practices. Race was collapsed to be Asian, Black, Multiracial, White, Other, and Missing/Unknown. Other includes American Indian, Other, and Pacific Islander. Ethnicity was categorized as Hispanic or non-Hispanic. Small for gestational age (SGA) was derived from documented birthweight using the Fenton growth chart [30]. Other diagnoses and outcomes were based on diagnosis codes, procedure codes, and facility charges. Diagnoses included: hydrops, atrioventricular septal defect (AVSD), atrial septal defect (ASD), ventricular septal defect (VSD), patent ductus arteriosus (PDA), pulmonary hypertension (PH), duodenal atresia/stenosis, and Hirschsprung’s disease. In-hospital medical utilization outcomes included: any central line, total parental nutrition (TPN), gastrostomy, mechanical ventilator, oscillator, noninvasive positive-pressure ventilation (NIPPV), high-flow nasal cannula (HFNC), and nitric oxide. In-hospital complications included: necrotizing enterocolitis (NEC) Bells 2 + , periventricular leukomalacia (PVL), interventricular hemorrhage or intracranial hemorrhage (IVH/ICH), retinopathy of prematurity (ROP), central-line associated bloodstream infection (CLABSI), and any infection. Disposition included death or discharge to hospice and unplanned readmission <30 days for those who survived to discharge.

### Statistical analysis

GA-based risk differences (RD) were examined for each diagnosis and outcome using linear probability models. Specifically, the risk of the given diagnosis or outcome among neonates in the given GA group (e.g., <28 weeks) was compared relative to full-term (i.e., 37+ week). For the outcome of readmission within 30 days, only neonates who did not die/were not discharged to hospice were included. Complete data were used for analysis and variables with counts <5 were excluded from analysis. We plotted RD estimates and corresponding 95% confidence intervals (CI); shading of the point estimate corresponds to GA group, with younger GA groups having a darker shade.

Data were cleaned using SAS version 9.4 and analyzed using R version 4.3.1. All tests were two-sided and alpha was set at 0.05. No adjustment for multiple tests was performed given the exploratory nature of the study.

## Results

A total of 8598 neonates were admitted to a participating PHIS hospital NICU < 30 days of age with the diagnosis of DS. Of those, 1503 neonates with DS were excluded due to missing GA. Another 58 neonates with DS had two NICU admissions <30 days of age and data were collapsed, resulting in 7037 neonates with DS admitted to a NICU < 30 days of age.

Demographics of our cohort are presented in Table 1. The extremely preterm group had the highest percentage of Black neonates at 19% (18/95) and Multiracial at 4% (4/95) and a lower percentage of White 51% (48/95) and Hispanic ethnicity at 21% (17/81) compared to the overall cohort. Additionally, area-level income was lower (dollars; median [interquartile range]; 37,356 [29,882,44,987] vs. 41,078 [33,204,52,509]) and government-only payment was higher (60% vs. 54%) in the extremely preterm cohort compared to overall.

**Table 1:** Demographics of neonates with down syndrome in the NICU stratified by gestational age group, 2008–2022.

	*n* (%) or median (25th–75th percentile)				
	Overall	<28 weeks	28–31 weeks	32–36 weeks	37+ weeks
n (%)	7037 (100)	95 (1)	304 (4)	2168 (31)	4470 (64)
Gestational age, in weeks	37 (36, 38)	26 (25, 27)	30 (29, 31)	35 (34, 36)	38 (37, 39)
Male sex	3921 (56)	54 (57)	165 (54)	1246 (58)	2456 (55)
Race					
Asian	221 (3)	3 (3)	10 (3)	69 (3)	139 (3)
Black	839 (12)	18 (19)	44 (15)	267 (12)	510 (11)
Multiracial	141 (2)	4 (4)	4 (1)	53 (2)	80 (2)
White	4403 (63)	48 (51)	184 (61)	1315 (61)	2856 (64)
Other^a^	1066 (15)	15 (16)	44 (15)	356 (16)	651 (15)
Missing/unknown	367 (5)	7 (7)	18 (6)	108 (5)	234 (5)
Ethnicity^b^					
Hispanic	1736 (27)	17 (21)	67 (25)	562 (28)	1090 (27)
Non-Hispanic	4662 (73)	64 (79)	207 (75)	1417 (72)	2974 (73)
Urbanicity^c^					
Rural	1228 (18)	19 (20)	50 (17)	365 (17)	794 (18)
Urban/suburban	5727 (82)	75 (80)	252 (83)	1782 (83)	3618 (82)
2010 zip code-level median household income, dollars	41,078 (33,204, 52,509)	37,356 (29,882, 44,987)	39,721 (32,382, 50,625)	41,733 (33,141, 52,554)	41,018 (33,319, 52,511)
Primary payor^d^					
Commercial only	2997 (43)	32 (36)	116 (38)	916 (42)	1933 (44)
Government only	3773 (54)	54 (60)	177 (58)	1194 (55)	2348 (53)
Other^e^	222 (3)	4 (4)	10 (3)	52 (2)	156 (4)

*NICU* neonatal intensive care unit.

^a^Includes Other, American Indian, and Pacific Islander.

^b^*n* = 639 missing ethnicity data.

^c^*n* = 82 missing urbanicity data.

^d^*n* = 45 missing primary payor data.

^e^Defined as non-commercial/government payor or >1 payor.

**Fig. 1:**
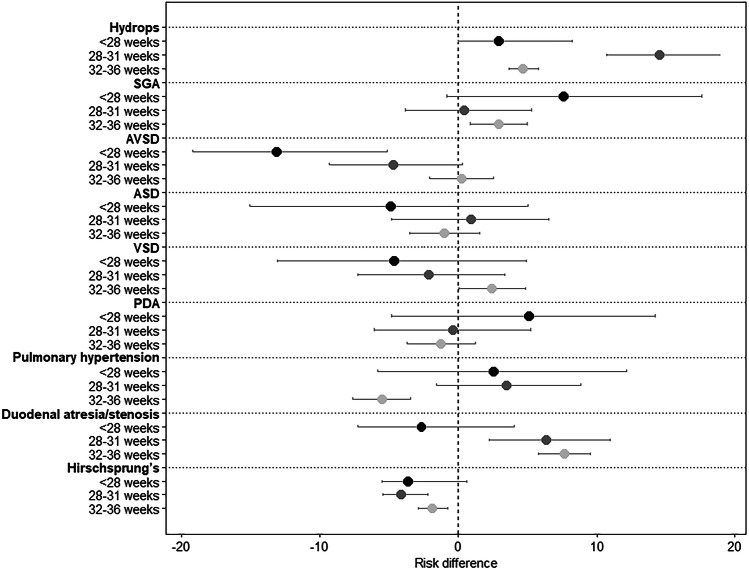
Risk differences of selected diagnoses for neonates with Down syndrome in the NICU by gestational age group (*N* = 7037; reference group: 37+ weeks). Shading of the point estimate represents a given gestational age group (e.g., <28 weeks) with darker shading corresponding to a younger gestational age group. Risk differences and 95% confidence intervals are presented as a percentage. NICU neonatal intensive care unit, SGA small for gestational age, AVSD atrioventricular septal defect, ASD atrial septal defect, VSD ventricular septal defect, PDA patent ductus arteriosus.

Figure 1 displays a forest plot of RD estimates for selected diagnoses for neonates with DS in the NICU by GA group in reference to term. Supporting data and RD estimates with 95% CI are provided in supplemental Tables 1, 2. Development of hydrops was higher in the very preterm (RD 14.5 [95% CI; 10.7, 18.9]) and moderate/late preterm (RD 4.7 [95% CI 3.7, 5.8]). That is, relative to term neonates, the risk of hydrops diagnosis was 14.5% higher for very preterm neonates and 4.7% higher for moderate/late preterm neonates. Moderate/late preterm neonates had a slightly higher risk of being SGA (RD 2.9, [95% CI; 0.8–5.0]). Atrioventricular septal defects were less prevalent in the extremely preterm cohort (RD –13.1, [95% CI –19.2, –5.2]). The rate of PH overall was 23% and risk was lower in the moderate/late preterm neonates (RD –5.6 [95% CI –7.6, –3.5]) but similar in the two most preterm groups compared to term. The risk for duodenal atresia/stenosis was higher in the very preterm (RD 6.3 [95% CI 2.2, 11.0]) and moderate/late preterm cohort (RD 7.6 [95% CI 5.8, 9.5]) whereas Hirschsprung’s disease was lower in these groups (RD –4.1 [95% CI –5.5, –2.2], RD –1.9 [95% CI –2.9, –0.8], for very preterm and moderate/late preterm groups, respectively).

**Fig. 2:**
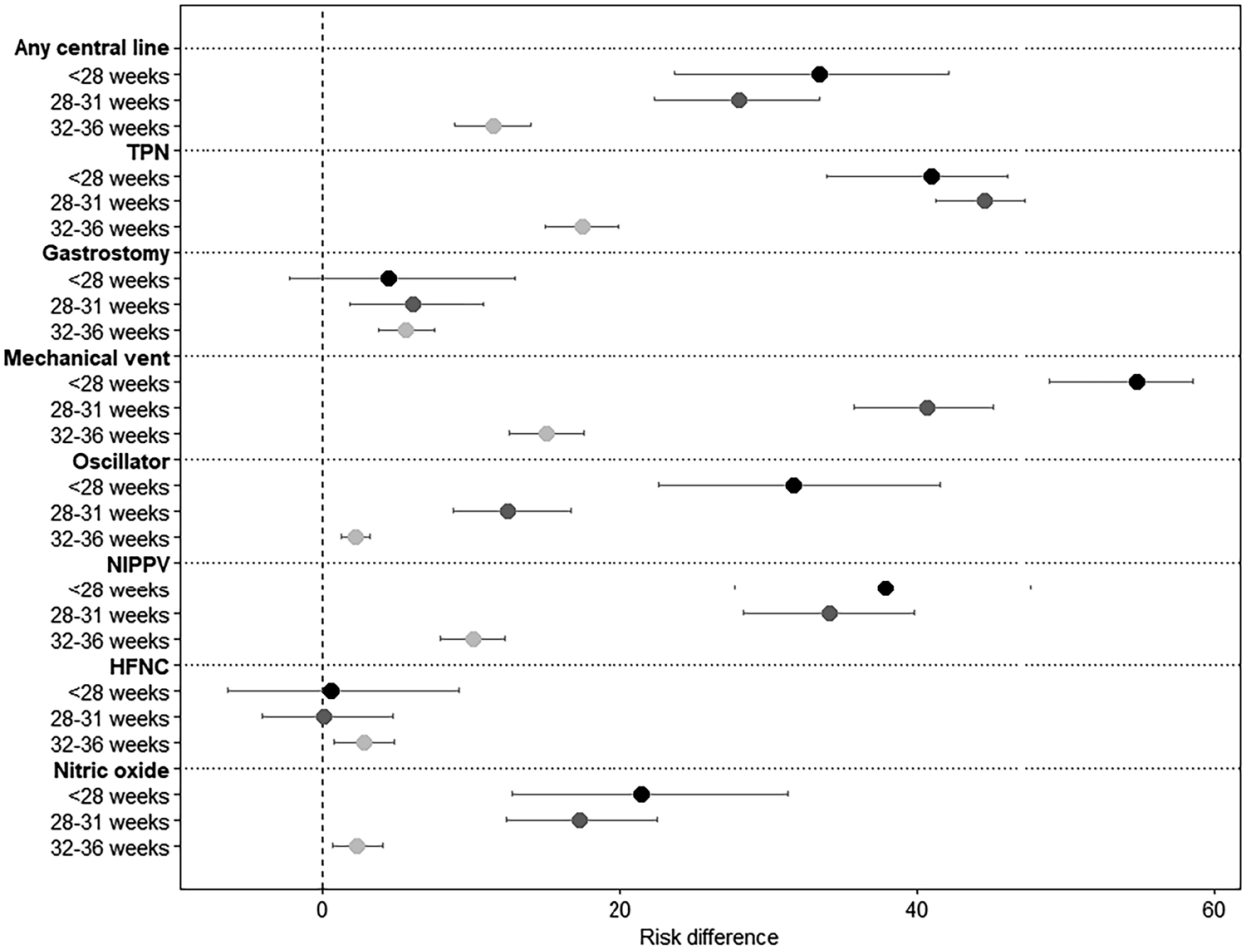
Risk differences of in-hospital medical utilization for neonates with down syndrome in the NICU by gestational age group (*N* = 7037; reference group: 37+ weeks). Shading of the point estimate represents a given gestational age group (e.g., <28 weeks) with darker shading corresponding to a younger gestational age group. Risk differences and 95% confidence intervals are presented as a percentage. NICU neonatal intensive care unit, TPN total parental nutrition, NIPPV noninvasive positive-pressure ventilation, HFNC high flow nasal cannula.

Figure 2 displays a forest plot of RD estimates for in-hospital medical utilization variables for neonates with DS in the NICU by GA group in reference to term. Among all neonates in our cohort, 42% required a central line and 47% required mechanical ventilation. Central line utilization, total parental nutrition administration, ventilator, oscillator, and nitric oxide usage were higher in all the preterm cohorts compared to the term cohort. Overall, 14% underwent gastrostomy tube placement. Gastrostomy tube placement was only slightly higher in the very preterm and moderate/late preterm cohorts (RD 6.1 [95% CI 1.9, 10.8] and RD 5.6 [95% CI 3.8, 7.5], respectively) but similar in the extremely preterm group compared to term. High-flow nasal cannula use was higher in the moderate/late preterm cohort compared to the term cohort (RD 2.8 [95% CI 0.8, 4.8]).

**Fig. 3:**
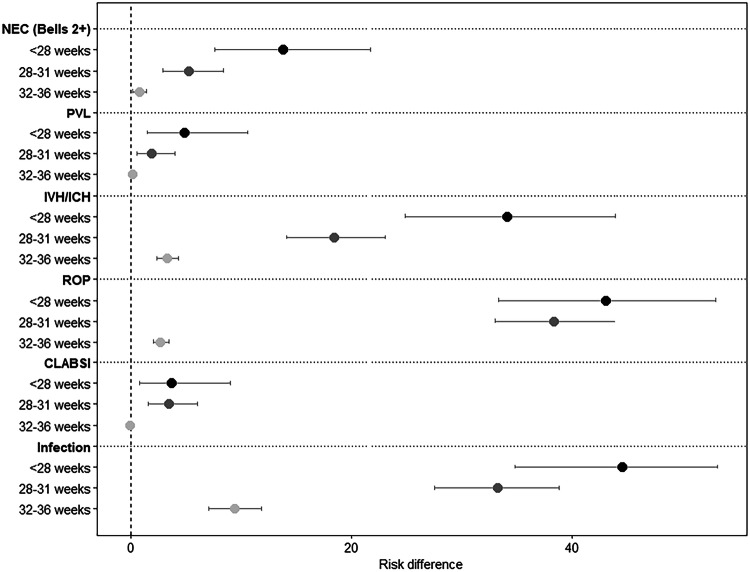
Risk differences of in-hospital complications for neonates with Down syndrome in the NICU by gestational age group (*N* = 7037; reference group: 37+ weeks). Shading of the point estimate represents a given gestational age group (e.g., <28 weeks) with darker shading corresponding to a younger gestational age group. Risk differences and 95% confidence intervals are presented as a percentage. NICU neonatal intensive care unit, NEC necrotizing enterocolitis, PVL periventricular leukomalacia, IVH interventricular hemorrhage, ICH intracranial hemorrhage, ROP retinopathy of prematurity, CLABSI central line-associated bloodstream infection.

Figure 3 displays a forest plot of RD estimates for in-hospital complications for neonates with DS in the NICU by GA group in reference to term. Complications commonly associated with prematurity, including risk of necrotizing enterocolitis, intraventricular hemorrhage, retinopathy of prematurity were inversely related to GA. Overall infection rate was high at 31%. There was a higher risk for PVL and CLABSI in the extremely preterm (RD 4.9 [95% CI 1.5, 10.6] and RD 3.7 [95% CI 0.8, 9.0]) and very preterm (RD 1.9 [95% CI 0.6, 4.0] and RD 3.5 [95% CI 1.6, 6.0]).

**Fig. 4:**
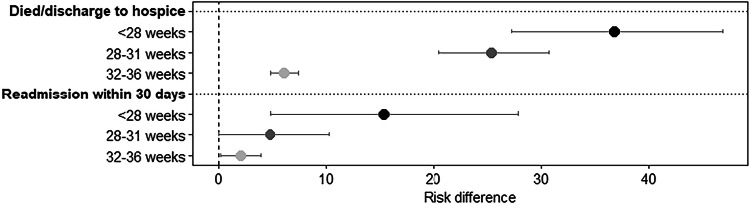
Risk differences of in-hospital mortality or readmission for neonates with Down syndrome in the NICU by gestational age group (*N* = 7037; reference group: 37+ weeks). Shading of the point estimate represents a given gestational age group (e.g., <28 weeks) with darker shading corresponding to a younger gestational age group. Risk differences and 95% confidence intervals are presented as a percentage. NICU, neonatal intensive care unit.

Figure 4 displays a forest plot of RD estimates for in-hospital mortality or unplanned readmission for neonates with DS in the NICU by GA group in reference to term. Readmission rates for those who survived to discharge increased as GA decreased (RD 2.1 [95% CI 0.2, 3.9], RD 4.8 [95% CI 0, 10.3], RD 15.4 [95% CI 4.8, 27.9] for moderate/late preterm, very preterm, and extremely preterm in reference to term, respectively). Overall, NICU mortality rate was 7% with progressively increasing mortality as GA decreased (RD 6.1 [95% CI 4.8, 7.5], RD 25.4 [95% CI 20.5, 30.6], RD 36.8 [95% CI 27.3, 46.8] for moderate/late preterm, very preterm and extremely preterm in reference to term, respectively).

## Discussion

This study examined a large, modern cohort of NICU patients with DS that received care across 47 freestanding US children’s hospitals. This study identified high rates of overall infection, feeding problems, mechanical ventilation, PH, and mortality in neonates with DS requiring intensive care. This study illustrates some demographic and socioeconomic differences in neonates with DS previously not explored. For those identified as Asian, multiracial, or “other” we did not appreciate significant differences in proportions by GA groups as opposed to White and Black. The higher percentage of Black neonates with DS born extremely premature is worth noting but may be confounded by the greater risk of preterm birth in non-Hispanic Black women compared to non-Hispanic White women [31]. Furthermore, Black neonates born very preterm have higher rates of morbidity and mortality compared to White neonates, which is important to consider in the context of this analysis [32]. GA-based differences in race in our cohort of neonates with DS is likely multifactorial, as is true in preterm neonates overall, and deserves further investigation. Extremely preterm neonates with DS were born to mothers residing in areas with lower income compared to older gestations, but again, this is not unique to neonates with DS. Higher socioeconomic status is associated with better perinatal outcomes including lower risk of preterm birth [33].

There were important differences with a decreased incidence of AVSD and Hirschsprung’s disease and an increased risk for duodenal atresia/stenosis in the more premature groups. The RD for PH was –5.6 (95% CI –7.6, –3.5) in the moderate/late preterm group, but not significantly different throughout the other GA groups, opposing the notion that this morbidity was related to prematurity alone. However, the risk of nitric oxide usage was higher with lower GA groups (RD 17.3 [95% CI 12.4, 22.5], RD 21.5 [95% CI 12.7, 31.3], very preterm and extremely preterm), potentially lending to the greater PH treatment needs seen with premature neonates with DS, recognizing they are at risk of bronchopulmonary dysplasia-associated PH in addition to the risk already known to be associated with DS [34, 35]. The etiology of PH in neonates with DS is thought to be multifactorial, including complex hemodynamics, altered lung development, and intrinsic endothelial dysfunction [36]. Despite increased prevalence, the rates of survival for DS-associated PH appear to be similar to non-DS associated PH [37]. However, those with respiratory complications may have decreased resolution [37].

Most medical utilization variables examined were expectedly more common in the most preterm groups. The association of earlier gestation with use of a central line and TPN is not surprising, as most institutions follow recommended guidelines for parental nutrition initiation in those born less than 31 weeks gestation [38]. Similarly, increasing prematurity is associated with higher risk of invasive respiratory support due to incomplete lung development, despite advances in ventilatory strategies [39, 40]. However, the similar risk of gastrostomy tube throughout GA groups may be emphasizing the feeding difficulties neonates with DS experience, irrespective of GA, as otherwise the risk would be highest in the extremely preterm neonates who have the longest delays in feeding initiation and progression [41]. One study by Poskanzer et al. highlighted the risk those with DS face of gastrostomy tube placement in the first year of life and did not find a difference in the rates between those with CHD or those without [42]. Further studies are needed to establish the best means of nutritional support in this population as optimal nutrition is vital for avoiding additional morbidities, such as aspiration pneumonia and poor growth.

The infection rate in this cohort was high at 31%. Previous studies of children with DS have shown they are highly susceptible to infections, particularly lower respiratory tract infections [43]. Moreover, children with DS and sepsis have a higher risk of mortality compared to those without DS [28]. Potential factors leading to increased risk and severity of infection in this population include: immunodeficiencies (i.e. T and B cell lymphopenia, impaired T cell proliferation, reduced antibody response, and neutrophil chemotaxis defects), metabolic and nutritional factors (i.e. Zinc deficiency), and abnormal anatomical structures that can increase susceptibility such as small ear canals and tracheomalacia [44, 45]. The higher risk of infection coupled with common lung disorders seen in those with DS, such as airway anomalies, interstitial disease, and sleep disordered breathing make neonates with DS more vulnerable to respiratory insufficiency/failure, necessitating greater breathing support [46]. It is feasible that this accounts for the high rates of unplanned readmission and need for respiratory support seen in our cohort, which was most notable in the extremely premature neonates with DS, a group also faced with premature lung disease. It is understandable that those born at earlier gestational age are at higher risk of morbidities related to prematurity and long term sequalae, leading to more complications and necessitating higher healthcare utilization after NICU discharge [47]. A study by Esperanza et al. examined hospital admissions of children with DS in Wales from 1990–2012 and found that of 356 neonates with DS, 80% had at least one hospital admission during the first year of life, with the most common reasons being congenital heart disease and respiratory infections [48]. The PHIS database differentiates between planned and unplanned readmissions, and it is plausible to assume that a high proportion of readmissions for congenital heart disease were planned (i.e., surgical repair) and thus, the unplanned readmissions seen in our cohort could be due to respiratory complications.

Overall NICU mortality rate of this cohort was 7% and mortality rates were highest in the most premature neonates with DS. It has been shown that mortality rates increase proportionally with decreasing GA or birthweight, however, for neonates with DS in the NICU a comprehensive understanding of specific causes of death are lacking, limiting our ability to implement targeted strategies for prevention and care [49]. Shimokaze et al. examined NICU mortality of infants with DS by GA over a 27-year period at a tertiary care center in Japan and found that bronchopulmonary dysplasia and PH were leading causes of death for very preterm and extremely preterm infants [50]. There is a need for more in-depth analysis of temporal trends and specific causes of death among neonates with DS in the United States.

There are multiple limitations to this study. First, this was a retrospective study using administrative data with inherent shortcomings of such a design. Data were limited by what was available via the PHIS database, which included only children’s hospitals. Since these hospitals are usually not birth hospitals, neonates were presumably transferred for specialized care, thus not all neonates with DS are represented. This cohort is not representative of all individuals with DS, as not all neonates with DS require NICU admission, or admission to a children’s hospital NICU where the severity of illness is typically higher, as is reflected in PHIS. Maternal information was not available given the nature of the data collection. In general, the severity of medical conditions could not be ascertained. The “infection” variable for PHIS included a broad range of diagnoses, which limited interpretability. Also, medical conditions are not able to be temporally related to mortality, thus cause and effect cannot be determined. Data were collected from a 14-year period over which advancements in care and modern management strategies may have affected the prevalence of some outcomes, which we were unable to control for during analysis. Since clinical information is primarily from ICD-9 and 10 codes, nonbillable data are likely to not be recorded. The dataset used for this study was unable to distinguish between the presence of a complete additional chromosome and partial translocations, limiting genotype-phenotype correlations. PHIS has a robust quality control procedure; however, we cannot verify that diagnoses were made and coded in a similar way across hospitals.

In conclusion, our study examined outcomes of a modern cohort including over 7000 neonates with DS requiring intensive care at over 45 children’s hospitals throughout the United States. Preterm birth strongly influenced rates of infection, hospital readmission, and mortality. Increasing prematurity predicts an overall higher rate of medical utilization including need for TPN, central line placement, nitric oxide, and respiratory support. Prenatal interventions for DS are needed that target the disproportionately higher rate of preterm birth that could lead to a decrease in their complications and utilization of medical care during the neonatal period.

## Supplementary information

Supplemental Tables 1 and 2

## Data Availability

The datasets generated during and/or analyzed during the current study are available from the corresponding author on reasonable request.
