# Supplementary material for: Gestational age-based outcomes of neonates with Down syndrome in the neonatal intensive care unit (NICU): review of pediatric health information system (PHIS) database
Source: J Perinatol. 2025 Aug 12;45(12):1751–7. doi: 10.1038/s41372-025-02384-0 (PMC12716992; doi:10.1038/s41372-025-02384-0)
Supplement: Supplementary file 1 — Supplemental Tables 1 and 2 [file 41372_2025_2384_MOESM1_ESM.docx]

**Supplemental Table 1:** Diagnoses, In-Hospital Medical Utilization, In-Hospital Complications, and Disposition of Neonates with Down Syndrome in the NICU by Gestational Age, 2008-2022

|  | **Overall**  **n (%)** | **<28 Weeks**  **n (%)** | **28-31 Weeks**  **n (%)** | **32-36 Weeks**  **n (%)** | **37+ Weeks**  **n (%)** |
| --- | --- | --- | --- | --- | --- |
| **n (%)** | 7,037 (100) | 95 (1) | 304 (4) | 2,168 (31) | 4,470 (64) |
| ***Diagnoses*** | | | | | |
| **Hydrops** | 238 (3) | 4 (4) | 48 (16) | 129 (6) | 57 (1) |
| **SGA^1^** | 1,147 (18) | 20 (24) | 46 (17) | 388 (19) | 693 (17) |
| **AVSD** | 1,864 (27) | 13 (14) | 67 (22) | 586 (27) | 1,198 (27) |
| **ASD** | 4,101 (58) | 51 (54) | 181(60) | 1,249 (58) | 2,620 (59) |
| **VSD** | 2,298 (33) | 26 (27) | 91 (30) | 748 (35) | 1,433 (32) |
| **PDA** | 4,284 (61) | 63 (66) | 185 (61) | 1,300 (60) | 2,736 (61) |
| **Pulmonary hypertension** | 1,638 (23) | 26 (27) | 86 (28) | 417 (19) | 1,109 (25) |
| **Duodenal atresia/stenosis** | 963 (14) | 8 (8) | 53 (17) | 406 (19) | 496 (11) |
| **Hirschsprung’s** | 351 (5) | 2 (2) | 5 (2) | 85 (4) | 259 (6) |
| ***In-Hospital Medical Utilization*** | | | | | |
| **Any central line** | 2,977 (42) | 67 (71) | 198 (65) | 1,053 (49) | 1,659 (37) |
| **TPN** | 4,040 (57) | 86 (91) | 286 (94) | 1,453 (67) | 2,215 (50) |
| **Gastrostomy** | 1,014 (14) | 16 (17) | 56 (18) | 390 (18) | 552 (12) |
| **Mechanical ventilator** | 3,315 (47) | 90 (95) | 245 (81) | 1,193 (55) | 1,787 (40) |
| **Oscillator** | 259 (4) | 32 (34) | 44 (15) | 92 (4) | 91 (2) |
| **NIPPV** | 1,549 (22) | 52 (55) | 155 (51) | 586 (27) | 756 (17) |
| **HFNC** | 1,280 (18) | 17 (18) | 53 (17) | 436 (20) | 774 (17) |
| **Nitric oxide** | 831 (12) | 30 (32) | 83 (27) | 269 (12) | 449 (10) |
| ***In-Hospital Complications*** | | | | | |
| **NEC (Bells 2+)** | 114 (2) | 14 (15) | 19 (6) | 38 (2) | 43 (1) |
| **PVL** | 41 (1) | 5 (5) | 7 (2) | 12 (1) | 17 (<1) |
| **IVH/ICH** | 278 (4) | 34 (36) | 61 (20) | 108 (5) | 75 (2) |
| **ROP** | 224 (3) | 41 (43) | 117 (39) | 61 (3) | 5 (<1) |
| **CLABSI** | 47 (1) | 4 (4) | 12 (4) | 9 (<1) | 22 (1) |
| **Infection** | 2,173 (31) | 67 (71) | 180 (59) | 767 (35) | 1,159 (26) |
| ***Disposition*** | | | | | |
| **Died/discharge hospice** | 473 (7) | 38 (40) | 87 (29) | 203 (9) | 145 (3) |
| **Readmission ≤ 30 days^2^** | 893 (14) | 16 (28) | 38 (18) | 290 (15) | 549 (13) |
| ^1^Derived from the Fenton growth curve using documented birthweight, n=474 missing data  ^2^ n=473 missing data  NICU, neonatal intensive care unit; SGA, small for gestational age; AVSD, atrioventricular septal defect; ASD, atrial septal defect; VSD, ventricular septal defect; PDA, patent ductus arteriosus; TPN, total parenteral nutrition; NIPPV, non-invasive positive pressure ventilation; HFNC, high-flow nasal cannula; NEC, necrotizing enterocolitis; PVL, periventricular leukomalacia; IVH, interventricular hemorrhage; ICH intracranial hemorrhage; ROP, retinopathy of prematurity; CLABSI, central line associated bloodstream infection | | | | | |

**Supplemental Table 2:** Risk Difference Estimates with 95% CI for Diagnoses, In-Hospital Medical Utilization, In-Hospital Complications, and Disposition of Neonates with Down Syndrome in the NICU (Reference Group: Neonates with Down Syndrome Born 37+ Weeks Gestational Age)

|  | **< 28 Weeks**  **n=95**  **RD (95% CI)** | **28-31 Weeks**  **n=304**  **RD (95% CI)** | **32-36 Weeks**  **n=2168**  **RD (95% CI)** |
| --- | --- | --- | --- |
| ***Diagnoses*** |  |  |  |
| **Hydrops** | 2.9 (0, 8.2) | 14.5 (10.7, 18.9) | 4.7 (3.7, 5.8) |
| **SGA*** | 7.6 (-0.8, 17.6) | 0.4 (-3.9, 5.3) | 2.9 (0.8, 5.0) |
| **AVSD** | -13.1 (-19.2, -5.2) | -4.8 (-9.4, 0.3) | 0.2 (-2, 2.5) |
| **ASD** | -4.9 (-15.1, 5) | 0.9 (-4.8, 6.5) | -1 (-3.5, 1.5) |
| **VSD** | -4.7 (-13.1, 4.9) | -2.1 (-7.3, 3.3) | 2.4 (0, 4.9) |
| **PDA** | 5.1 (-4.8, 14.2) | -0.4 (-6.1, 5.2) | -1.2 (-3.8, 1.3) |
| **Pulmonary hypertension** | 2.6 (-5.8, 12.1) | 3.5 (-1.6, 8.9) | -5.6 (-7.6, -3.5) |
| **Duodenal atresia/stenosis** | -2.7 (-7.3, 4.1) | 6.3 (2.2, 11) | 7.6 (5.8, 9.5) |
| **Hirschsprung’s** | -3.7 (-5.6, 0.6) | -4.1 (-5.5, -2.2) | -1.9 (-2.9, -0.8) |
| ***In-Hospital Medical Utilization*** |  |  |  |
| **Any central line** | 33.4 (23.7, 42.1) | 28 (22.4, 33.4) | 11.5 (8.9, 14) |
| **TPN** | 41 (33.9, 46) | 44.5 (41.2, 47.3) | 17.5 (15, 19.9) |
| **Gastrostomy** | 4.5 (-2.2, 12.9) | 6.1 (1.9, 10.8) | 5.6 (3.8, 7.5) |
| **Mechanical ventilator** | 54.8 (48.9, 58.5) | 40.6 (35.7, 45) | 15.1 (12.5, 17.6) |
| **Oscillator** | 31.6 (22.7, 41.5) | 12.4 (8.8, 16.7) | 2.2 (1.3, 3.2) |
| **NIPPV** | 37.8 (27.7, 47.7) | 34.1 (28.4, 39.8) | 10.1 (8, 12.3) |
| **HFNC** | 0.6 (-6.3, 9.2) | 0.1 (-4, 4.8) | 2.8 (0.8, 4.8) |
| **Nitric oxide** | 21.5 (12.7, 31.3) | 17.3 (12.4, 22.5) | 2.4 (0.7, 4) |
| ***In-Hospital Complications*** |  |  |  |
| **NEC (Bells 2+)** | 13.8 (7.6, 21.8) | 5.3 (2.9, 8.4) | 0.8 (0.2, 1.5) |
| **PVL** | 4.9 (1.5, 10.6) | 1.9 (0.6, 4) | 0.2 (-0.2, 0.6) |
| **IVH/ICH** | 34.1 (24.9, 44) | 18.4 (14.1, 23.1) | 3.3 (2.4, 4.3) |
| **ROP** | 43 (33.4, 53.1) | 38.4 (33, 43.9) | 2.7 (2.1, 3.5) |
| **CLABSI** | 3.7 (0.8, 9) | 3.5 (1.6, 6) | -0.1 (-0.4, 0.3) |
| **Infection** | 44.6 (34.9, 53.2) | 33.3 (27.6, 38.9) | 9.4 (7.1, 11.8) |
| ***Disposition*** |  |  |  |
| **Died/discharge hospice** | 36.8 (27.3, 46.8) | 25.4 (20.5, 30.6) | 6.1 (4.8, 7.5) |
| **Readmission ≤ 30 days** | 15.4 (4.8, 27.9) | 4.8 (0, 10.3) | 2.1 (0.2, 3.9) |
| *Derived from the Fenton growth curve using documented birthweight  CI, confidence interval; NICU, neonatal intensive care unit; RD, risk difference estimate; SGA, small for gestational age; AVSD, atrioventricular septal defect; ASD, atrial septal defect; VSD, ventricular septal defect; PDA, patent ductus arteriosus; TPN, total parenteral nutrition; NIPPV, non-invasive positive pressure ventilation; HFNC, high-flow nasal cannula; NEC, necrotizing enterocolitis; PVL, periventricular leukomalacia; IVH, interventricular hemorrhage; ICH intracranial hemorrhage; ROP, retinopathy of prematurity; CLABSI, central line associated bloodstream infection | | | |
